# 18 Comparison of Topical Agents for Eschar Removal in Porcine Model: Bromelain-enriched vs. Traditional Collagenase Agents

**DOI:** 10.1093/jbcr/irac012.022

**Published:** 2022-03-23

**Authors:** Adam Singer, Samuel Grandfield, Eshani Goradia, Kunal Shah, Nigel Zhang, Steve McClain

**Affiliations:** Stony Brook University Medical Center, Stony Brook, New York; Stony Brook University Medical Center, Stony Brook, New York; Stony Brook University Medical Center, Stony Brook, New York; Stony Brook University Medical Center, Stony Brook, New York; Stony Brook University Medical Center, Stony Brook, New York; Stony Brook University Medical Center, Stony Brook, New York

## Abstract

**Introduction:**

Surgical excision and grafting of deep partial thickness (DPT) and full thickness (FT) burns is a cornerstone of modern burn wound care. Use of commercially available topical enzymatic agents has been limited due to slower and less complete eschar removal than surgical excision. Using a porcine model of DPT and FT burns, we compared the eschar removal efficacy of a bromelain-enriched enzymatic agent derived from the stems of pineapple plants and a commercially available collagenase.

**Methods:**

Under an approved animal welfare committee protocol, we created 10 DPT and 10 FT burns measuring 2.5 by 2.5 cm on each of four anesthetized Yorkshire pigs weighing 30 kg using a preheated aluminum bar applied to the dorsum and flanks of the animals. For DPT burns, the bar was preheated to 80ºC and applied for 20 seconds with a pressure of 2 kg. For FT burns, the bar was preheated to 100ºC and applied for 30 seconds. Eschar removal was initiated 24-hours later. Two pigs each were randomly assigned to collagenase or the bromelain-enriched agent. The bromelain-enriched agent was applied topically once on day 1 for a period of 4 hours followed by a 2-hour soaking period with normal saline. The collagenase was applied topically daily as per manufacturer instructions until complete removal of the eschar or for up to 14 days. The primary outcome was the percentage of burns with complete eschar removal assessed by a masked observer at 1, 10 and 14 days after application.

**Results:**

A total of 40 FT and 40 DPT burns were created on the four pigs. Regarding FT burns, all bromelain treated burns experienced complete removal of eschar on day 1 after the single 4-hour application. In contrast, none of the collagenase treated FT burns experienced complete removal of eschar after 14 days of treatment. At 10 days, 30% of collagenase treated burns had < 50% removed eschar; by day 14, 40% had >50% removed eschar and 15% had < 50% eschar removed. Regarding DPT burns, all bromelain treated burns had complete eschar removal after the single 4 hour application. In contrast, none of the collagenase treated burns experienced complete removal of eschar after 10 days; by day 14, 35% had complete eschar removal. At day 10, 10% of collagenase treated burns had >50% eschar removed and 75% had < 50% eschar removed; by day 14, 30% had > 50% eschar removed and 35% had < 50% eschar removed. There were no wound infections or any other adverse events noted.

**Conclusions:**

Topical treatment of DPT and FT porcine burns with a single 4-hour application of a bromelain-enriched enzyme resulted in complete eschar removal of all burns. In contrast, after 14 daily topical applications of the collagenase, none of the FT burns experienced complete removal of the eschar and in 35% of the DPT burns, eschar removal was complete.

